# Anti-Müllerian hormone (AMH) in the Diagnosis of Menstrual Disturbance Due to Polycystic Ovarian Syndrome

**DOI:** 10.3389/fendo.2019.00656

**Published:** 2019-09-26

**Authors:** Ali Abbara, Pei Chia Eng, Maria Phylactou, Sophie A. Clarke, Tia Hunjan, Rachel Roberts, Sunitha Vimalesvaran, George Christopoulos, Rumana Islam, Kate Purugganan, Alexander N. Comninos, Geoffrey H. Trew, Rehan Salim, Artsiom Hramyka, Lisa Owens, Tom Kelsey, Waljit S. Dhillo

**Affiliations:** ^1^Department of Investigative Medicine, Imperial College London, Hammersmith Hospital, London, United Kingdom; ^2^Hammersmith In Vitro Fertilisation Unit, Imperial College Healthcare NHS Trust, London, United Kingdom; ^3^School of Computer Science, University of St. Andrews, St. Andrews, United Kingdom; ^4^Institute of Reproductive and Developmental Biology, Imperial College London, Hammersmith Hospital, London, United Kingdom

**Keywords:** polycystic ovarian syndrome (PCOS), antral follicle count (AFC), Anti-Müllerian hormone (AMH), amenorrhea, oligomenorrhea, hyperandrogenism

## Abstract

**Introduction:** Polycystic ovarian syndrome (PCOS) is a leading cause of female subfertility worldwide, however due to the heterogeneity of the disorder, the criteria for diagnosis remains subject to conjecture. In the present study, we evaluate the utility of serum Anti-Müllerian hormone (AMH) in the diagnosis of menstrual disturbance due to PCOS.

**Method:** Menstrual cycle length, serum AMH, gonadotropin and sex-hormone levels, total antral follicle count (AFC), body mass index (BMI) and ovarian morphology on ultrasound were analyzed in a cohort of 187 non-obese women, aged 18–35 years, screened for participation in a clinical trial of fertility treatment between 2013 and 2016 at a tertiary reproductive endocrine center.

**Results:** Serum AMH was higher in women with menstrual disturbance when compared to those with regular cycles (65.6 vs. 34.8 pmol/L; *P* < 0.0001). The odds of menstrual disturbance was increased 28.5-fold (95% CI 3.6–227.3) in women with serum AMH >60 pmol/L, in comparison to those with an AMH < 15 pmol/L. AMH better discriminated women with menstrual disturbance (area under ROC 0.77) from those with regular menstrual cycles than AFC (area under ROC 0.67), however the combination of the two markers increased discrimination than either measure alone (0.83; 95% CI 0.77–0.89). Serum AMH was higher in women with all three cardinal features of PCOS (menstrual disturbance, hyperandrogenism, polycystic ovarian morphology) when compared to women with none of these features (65.6 vs. 14.6 pmol/L; *P* < 0.0001). The odds of menstrual disturbance were increased by 10.7-fold (95% CI 2.4–47.1) in women with bilateral polycystic morphology ovaries than those with normal ovarian morphology. BMI was a stronger predictor of free androgen index (FAI) than either AMH or AFC.

**Conclusion:** Serum AMH could serve as a useful biomarker to indicate the risk of menstrual disturbance due to PCOS. Women with higher AMH levels had increased rates of menstrual disturbance and an increased number of features of PCOS.

## Introduction

Polycystic Ovarian Syndrome (PCOS) is a leading cause of anovulatory infertility, which affects up to 21% of women of reproductive age (1, 2). PCOS is characterized by a spectrum of signs and symptoms encompassing androgen excess, ovulatory disruption, polycystic ovarian (PCO) morphology and metabolic abnormalities, however no single feature is requisite for the diagnosis (1–3). Therefore, the diagnosis of PCOS can be challenged by the heterogeneity and complexity of its phenotypic presentation (4, 5).

The Rotterdam criteria is most widely-used for the diagnosis of PCOS, requiring the presence of two or more of the following features: oligo/amenorrhea, clinical or biochemical hyperandrogenism, and PCO morphology on ultrasound (6). More recently, international guidelines have updated the criteria for diagnosis of PCOS (2). A major feature of the diagnosis of PCOS is the presence of PCO morphology on ultrasound, which describes the typical peripheral pattern of follicular distribution in the ovary around a central stroma (7, 8). However, due to the subjective interpretation of this feature, PCO morphology is more commonly defined by follicle number per ovary (FNPO) (1, 2). Whereas, the Rotterdam criteria defined PCO morphology by the presence of at least 12 FNPO, due to advances in ultrasound resolution these criteria have been revised to be at least 20 FNPO in the updated guidelines (2).

AMH is a member of the transforming growth factor-β superfamily that is produced by growing ovarian antral follicles (1, 2, 9). Serum Anti-Müllerian hormone (AMH) correlates with the total number of antral follicles over both ovaries, and therefore has been proposed as a biomarker for PCOS diagnosis (9, 10). AMH has the advantage of being non-invasive and is relatively stable across the menstrual cycle, whereas AFC and ovarian morphology are best assessed during the follicular phase (9). However, due to the variability in levels using older less reliable assays (11) and the lack of an international standard (9, 12), AMH has yet to be adopted as part of the diagnostic criteria for PCOS (2).

More recently, an emerging hypothesis proposes that AMH may play a key role in the pathogenesis of PCOS as an endocrine signal rather than being merely a marker of ovarian follicle count (13, 14). A subset of gonadotropin releasing hormone (GnRH) neurons express the AMH receptor, and administration of AMH stimulates GnRH neuronal firing (15). Pituitary gonadotropes are sensitive to alterations in GnRH pulsatility, such that increased GnRH pulsatility is associated with LH-predominant gonadotropin secretion, which is a characteristic, albeit not universal, feature of PCOS (16, 17). Women with PCOS have higher levels of AMH than matched controls (18, 19). AMH levels fall with increasing age, which is concurrent with an improvement in some clinical features of PCOS (9, 20). For example, women with PCOS who are anovulatory may normalize their menstrual cycles with increased age (21). Indeed, cycle length shortens with increasing age prior to the climacteric even in women without PCOS, corresponding to a fall in ovarian reserve markers (9, 22).

In the present study, we analyzed serum AMH and ovarian morphology on ultrasound, in a cohort of 187 non-obese young women screened for participation in a clinical trial of fertility treatment between 2013 and 2016 to evaluate the utility of serum AMH in the prediction of menstrual disturbance due to PCOS.

## Materials and Methods

### Study Participants

Data was obtained from women screened for participation in a clinical trial of the novel trigger kisspeptin during IVF treatment conducted between 2013 and 2016 (23–25). The trial was registered on the National Institutes of Health Clinical Trials database (NCT01667406) and was approved by the Hammersmith and Queen Charlotte's Research Ethics Committee, London, UK (reference: 10/H0707/2). All recruited participants provided written informed consent prior to inclusion in the study with the Declaration of Helsinki. Inclusion criteria for the trial were women aged 18–35 years with both ovaries intact, BMI 18–30 kg/m^2^, serum AMH more than 10 pmol/L. Women with moderate/severe endometriosis or poor response to or >2 previous cycles of IVF treatment were excluded.

Clinical, biochemical and sonographic features of PCOS were analyzed to determine the relationship between serum AMH level, menstrual disturbance, total AFC on ultrasound, body mass index (BMI), serum gonadotropin levels, and androgen levels. During screening, women were clinically assessed by experienced physicians and provided a detailed menstrual cycle history, including number of menses per year, age of menarche, duration of menstrual cycle, previous use of hormonal contraceptive, as well as smoking history, medical conditions, medication use and duration of infertility. Oligomenorrhea was defined as menstrual cycle intervals ≥35 days (or 4–8 periods per year), whereas amenorrhea was defined as ≤ 3 menses per year. Biochemical evaluation included serum luteinizing hormone, LH; follicle stimulating hormone, FSH, testosterone, estradiol, and progesterone, sex hormone–binding globulin (SHBG), serum AMH measurements and free androgen index (FAI) (total testosterone in nmol/L × 100/SHBG in nmol/L). Total antral follicle count (AFC) and serum AMH were measured during the follicular phase (days 1–5) of the menstrual cycle. A transvaginal pelvic ultrasound was performed by one of three specialist sonographers using a 7.5 MHz transvaginal probe (Toshiba Xario Prime, Crawley, UK). The number of antral follicles (2–10 mm) per ovary (FNPO), was measured in the maximum plane section of both ovaries. In addition to classification by FNPO, morphological appearance was also classified as being polycystic ovarian (PCO) morphology if follicles were peripherally distributed around a central stroma or multicystic ovarian (MCO) morphology if ovaries had an increased number of follicles that were uniformly distributed.

### Assays

Serum LH, FSH, oestradiol, and testosterone were measured using automated chemiluminescent immunoassays (Abbott Diagnostics, Maidenhead, UK). Interassay coefficients of variations were as follow: LH 3.4%, FSH 3.5%; oestradiol, 3.4%; testosterone 3.6%. Limits of detectability for each assay are as follows LH 0.07 mIU/mL; FSH 0.05 mIU/mL; oestradiol 70 pmol/L (19 pg/mL); testosterone 0.08 nmol/L. AMH was measured using an enzyme linked immunosorbent assay (Beckman Coulter Inc, Brea, CA, USA). The reference range was 2.2–48.5 pmol/L (0.3–6.8 ng/mL) with a lower limit of detection of 0.6 pmol/l (0.08 ng/mL). The upper limit of detection for the AMH assay was 68.9 pmol/L. Testosterone is measured first-line by immunoassay and all elevated female testosterone results >2.0 nmol/L are reflexed for confirmation by LC-MS/MS. The interassay coefficient of variation was 4.6%; the intraassay coefficient of variation was 4.0%.

### Statistical Analysis

Analyses were conducted using Graphpad Prism version 7.0 and Stata Version 14.0 statistical software. Relative predictive performance analysis by random forest analysis and ROC curve analysis was performed in R version 3.5.1. Continuous variables that were parametrically distributed were reported by mean ± standard deviation (SD), whereas skewed continuous variables were summarized using median ± interquartile range (IQR). Parametrically distributed variables were compared using unpaired student's *t*-test (two groups) or one-way ANOVA (multiple groups). Non-parametrically distributed variables were compared using Kruskal-Wallis test or Mann Whitney as appropriate. Categorical data were compared using X^2^ or logistic regression.

## Results

### Baseline Characteristics

Of 207 women who were screened for the parent study, 187 for whom data was available were included in the present study. Baseline characteristics of the 187 included participants are summarized in Table 1. Oligo/amenorrhea was present in one third of women; women with oligo/amenorrhea had higher BMI (25.0 vs. 23.3 kg/m^2^), AMH (65.6 vs. 34.8 pmol/L), AFC (38 vs. 29), and FAI (3.0 vs. 2.1%) (Table 1).

**Table 1:** Baseline characteristics of patients with oligo/amenorrhea and women with regular cycles.

	**Eumenorrhea**	**Oligo/Amenorrhea**	**Total**	***P*-value**
No (%)	126 (67.4%)	61 (32.7%)	187 (100%)	
Ethnicity (%)				
Asian	33 (26.2)	20 (32.8)	53 (28.3)	
Black	7 (5.55)	3 (4.92)	10 (5.35)	
White	75 (59.6)	33 (54.1)	108 (57.8)	
Other	12 (9.52)	4 (6.56)	16 (8.56)	
Age (years)	31.0 (29.0, 33.0)	30.0 (28.0, 32.9)	31.0 (28.4, 33.0)	0.34
Age at menarche (years)	12.2 (11.5, 13.2)	13.0 (11.3, 13.0)	12.4 (11.3, 14.0)	0.28
BMI (kg/m^2^)	23.3 (21.7, 23.7)	25.0 (22.3, 28.4)	23.7 (21.8, 26.9)	0.02
Total antral follicle count	29 (24,37)	38 (28, 50)	30 (25,44)	<0.001
Ovarian volume (ml)	6.0 (2.5, 10.2)	6.9 (3.2, 12)	8.3 (6.1, 10.5)	0.01
Anti-Mullerian hormone (pmol/L)	34.6 (20.7, 52.2)	65.8 (45.8, 69.0)	45.0 (25.7, 69)	<0.001
Androstenedione (nmol/L)^*^	4.8 (3.8, 6.5)	4.0 (3.2, 5.5)	4.4 (3.6, 6.2)	0.06
Testosterone (nmol/L)	1.2 (0.9, 1.6)	1.4 (1.1, 1.8)	1.3 (1, 1.6)	0.03
Dehydroepiandrostenedione (DHEA) (μmol/L)^**^	5.4 (4.2, 9.4)	5.5 (4.4, 6.9)	5.5 (4.3, 7.0)	0.94
Free androgen index (FAI)	2.1% (1.5%, 4.0%)	3.0% (1.9%, 5.1%)	2.5% (1.6%, 4.5%)	0.02

*Median and interquartile range (IQR) are presented. Groups were compared by Mann-Whitney U test*.

* *Data was available for 35 eumenorrheic women and 40 oligo/amenorrheic women*.

** *Data was available for 29 eumenorrheic women and 35 oligo/amenorrheic women*.

### Clinical Features of PCOS and AMH/AFC

**Figure 1:**
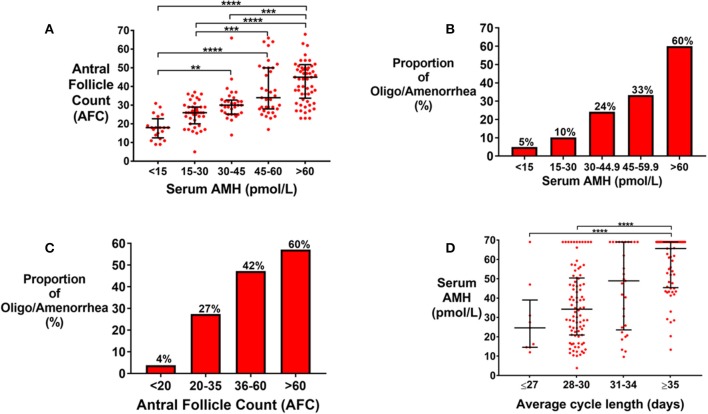
**(A)** Scattergram (median ± IQR) of serum anti-Mullerian hormone, AMH (pmol/L), by total antral follicle count (AFC) across both ovaries. Groups were compared by Kruskal Wallis test with *post-hoc* Dunn's multiple comparison test. AFC was significantly higher by category of serum AMH. **P < 0.01, ***P < 0.001, ****P < 0.0001. **(B)** Frequency of oligomenorrhea was increased by category of serum AMH (pmol/L) when compared by univariate logistic regression (*P* < 0.0001). The sample size in each AMH category is as follows: <15 pmol/L (*n* = 20); 15–30 pmol/L (*n* = 39), 30–44.9 pmol/L (*n* = 33), 45–59.9 (*n* = 33), >60 pmol/L (*n* = 60). The odds of oligomenorrhea was increased 2.2-fold (95% CI 0.2–20.8) in those with AMH 15–30 pmol/L; 6.1-fold (95% CI 0.70–52.9) in those with AMH 30–44.9 pmol/L; 9.5-fold (95% CI 1.1–80.5) in those with AMH 45–59.9 pmol/L; and 28.5-fold (95% CI 3.6–227.3) in those with AMH >60 pmol/L, compared to those with AMH < 15 pmol/L. **(C)** Frequency of oligomenorrhea was increased by categories of AFC when compared by univariate logistic regression (*P* < 0.0001). The number of patients in each category is as follows: AFC < 20 (*n* = 26); 20–35 (*n* = 91); 36–60 (*n* = 55), >60 (*n* = 15). The odds of oligomenorrhea was increased 9.5-fold (95% CI 1.2–73.6) in those with AFC 20–35; 22.4-fold (95% CI 2.8–177.2) in those with AFC 36–60; and 37.5-fold (95% CI 3.6–332) in those with AFC >60 when compared to those with AFC < 20. **(D)** Scattergram (median ± IQR) of serum AMH (pmol/L) by average menstrual cycle lengths. Groups were compared by the Kruskal Wallis test with *post-hoc* Dunn's multiple comparisons test. Women with average menstrual cycle length of ≥35 days have higher AMH levels compared to those with menstrual cycle length of ≤ 27 days (*P* < 0.0001) or those with menstrual cycle length of 28–30 days. ****P < 0.0001.

Total AFC on ultrasound was increased by category of serum AMH (Figure 1A). Oligo/amenorrhea was more frequent by increasing category of AMH (Figure 1B), or AFC (Figure 1C). The odds of oligo/amenorrhea were increased 28.5-fold (95% CI 3.6–227.3) in women with AMH >60 pmol/l, when compared to those with AMH < 15 pmol/L (Figure 1B). There was a trend toward higher AMH levels in patients with longer self-reported menstrual cycle length even in eumenorrheic women with cycle lengths <35 days (Figure 1D).

**Figure 2:**
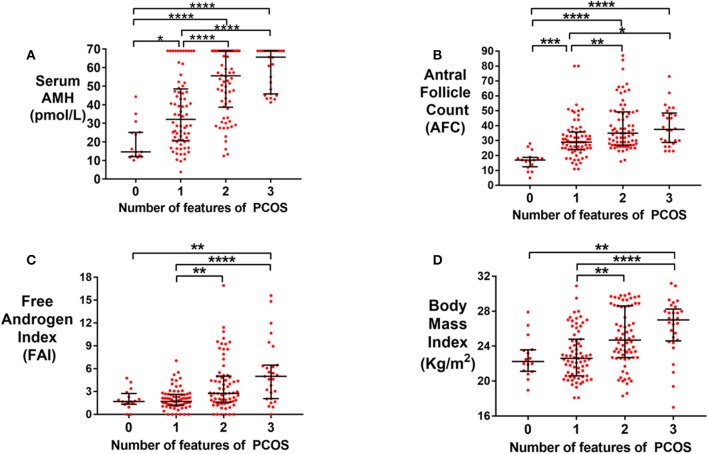
**(A)** Scattergram (median ± IQR) of serum AMH (pmol/L) by the number of features of PCOS (Rotterdam criteria). Groups were compared by the Kruskal Wallis test with *post-hoc* Dunn's multiple comparison test. Serum AMH was increased by the number of features of PCOS. *P < 0.05, **P < 0.01, ***P < 0.001, ****P < 0.0001. **(B)** Scattergram (median ± IQR) of total antral follicle count (AFC) in women by the number of features of PCOS (Rotterdam criteria). Groups were compared by Kruskal Wallis test with *post-hoc* Dunn's multiple comparison test. AFC was increased by the number of features of PCOS. *P < 0.05, **P < 0.01, ***P < 0.001, ****P < 0.0001. **(C)** Scattergram (median ± IQR) of Free Androgen Index, FAI, in women by the number of features of PCOS (Rotterdam criteria). Groups were compared by the Kruskal Wallis test with *post-hoc* Dunn's multiple comparison test. FAI was increased by the number of features of PCOS. **P < 0.01, ***P < 0.001. **(D)** Scattergram (median ± IQR) of Body Mass Index, BMI (kg/m^2^), in women by the number of features of PCOS (Rotterdam criteria). Groups were compared by the Kruskal Wallis test with *post-hoc* Dunn's multiple comparison test. BMI was increased by the number of features of PCOS. **P < 0.01, ***P < 0.001.

Serum AMH was higher in patients with more features consistent with PCOS (menstrual disturbance, hyperandrogenism, PCOM by Rotterdam criteria) (Figure 2A). Women with all three features had a significantly higher median serum AMH compared to those with none of these features (65.6 vs. 14.6 pmol/L; *P* < 0.0001). Similarly, median AFC (Figure 2B), free androgen index (FAI; Figure 2C) and BMI (Figure 2D) were also higher in women with all three features consistent with PCOS compared to women with none of these features. Results were similar when PCO morphology was categorized as per the new international PCOS guidelines (Supplementary Figure 1) (2).

**Figure 3:**
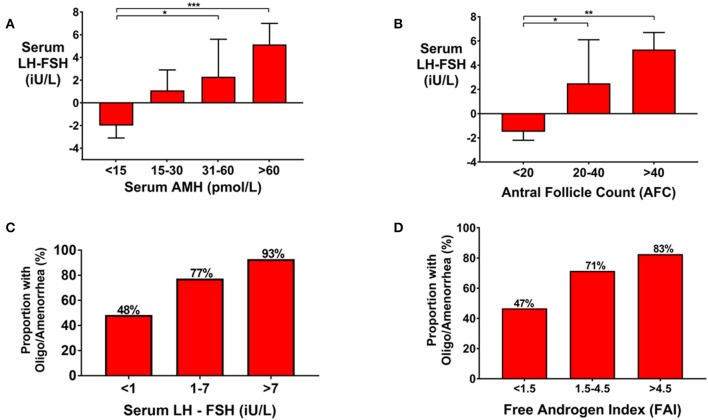
**(A)** Bar graph (Median ± IQR) of serum LH-FSH (iU/L) by categories of serum AMH (pmol/L) in women presenting with oligo/amenorrhea, or eumenorrheic women in the follicular phase of the menstrual cycle (days 1–8). Groups were compared by the Kruskal Wallis test with *post-hoc* Dunn's multiple comparison test. Gonadotropin secretion was more LH-predominant with increasing category of AMH. *P < 0.05, ***P < 0.001. **(B)** Bar graph (median ± IQR) of serum LH-FSH (iU/L) by categories of AFC in women presenting with oligo/amenorrhea or eumenorrheic women in the follicular phase of the menstrual cycle (days 1–8). Groups were compared by the Kruskal Wallis test with *post-hoc* Dunn's multiple comparison test. Gonadotropin secretion was more LH-predominant with increasing category of AFC. *P < 0.05, **P < 0.01. **(C)** Frequency of oligomenorrhea was higher by increasing categories of serum LH-FSH (iU/L) when compared by univariate logistic regression (*P* = 0.003). The number of women in each category were as follows: serum LH-FSH < 1 iU/L (*n* = 29); 1–7 iU/L (*n* = 44), >7 iU/L (*n* = 14). The odds of oligomenorrhea was increased by 3.6-fold (95% CI 1.3–10.0) in those with serum LH-FSH 1–7 iU/L and by 13.9-fold (95% CI 1.6–121) in those with serum LH-FSH >7 iU/L compared to those with serum LH-FSH < 1 iU/L. **(D)** The frequency of oligomenorrhea were higher by category of Free Androgen Index (FAI). The number of women in each categories of FAI were as follows: FAI < 1.5 (*n* = 34); 1.5–4.5 (*n* = 94); >4.5 (*n* = 44). When compared by univariate logistic regression, the odds of oligomenorrhea was increased 2.9-fold (95% CI 1.0–16.8) in those with FAI > 4.5 compared to those with FAI < 1.5 (*P* = 0.039).

Follicular phase serum gonadotropin levels were progressively more LH-predominant (consistent with increased GnRH pulsatility) by increasing categories of serum AMH (Figure 3A), or AFC (Figure 3B). Oligo/amenorrhea was more frequent in women with more LH-predominant gonadotropin secretion (Figure 3C), or higher FAI levels (Figure 3D). The odds of oligo/amenorrhea were increased by 13.9-fold (95% CI 1.6–121) in women with serum LH-FSH exceeding 7 iU/L when compared with those with a value <1 iU/L (*P* = 0.017) (Figure 3C). The odds of oligo/amenorrhea for women with an FAI>4.5 was increased by 2.9-fold (95% CI 1.0–16.8) compared to those with an FAI < 1.5 (*P* = 0.039) (Figure 3D).

### Oligo/Amenorrhea

**Figure 4:**
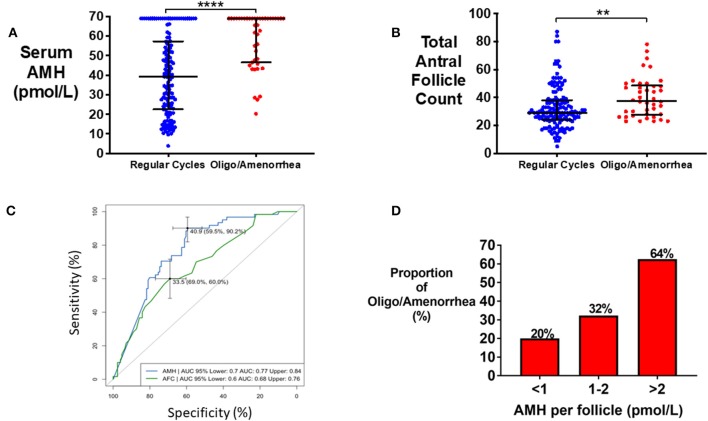
**(A)** Scattergram (median ± IQR) of serum AMH (pmol/L) in eumenorrheic women and in women with oligo/amenorrhea. Groups were compared by the Mann-Whitney *U* test. Women with oligomenorrhea/amenorrhea had statistically significant higher serum AMH. ****P < 0.0001. **(B)** Scattergram (median ± IQR) of total AFC in eumenorrheic women and in women with oligo/amenorrhea. Groups were compared by the Mann-Whitney test. Women with oligo/amenorrhea had higher AFC than eumenorrheic women. **P < 0.01. **(C)** Receiver Operating Characteristic (ROC) curves for the prediction of oligo/amenorrhea using serum AMH (pmol/L) and AFC. **(D)** Frequency of oligomenorrhea increased with category of AMH per antral follicle (serum AMH value divided by total antral follicle count) when compared by univariate logistic regression (*P* = 0.001). The odds of oligomenorrhea was increased by 1.9-fold (95% CI 0.9–4.1) in those with AMH per antral follicle 1–2 pmol/L, and 6.7-fold (95% CI 2.5–19.9) in those with AMH per antral follicle >2 pmol/L compared to those with an AMH per antral follicle <1 pmol/L.

Median serum AMH was significantly higher in women with oligo/amenorrhea when compared to women with regular menstrual cycles (65.6 vs. 34.8 pmol/L; *P* < 0.0001) (Figure 4A). Total AFC was also higher in women with oligo/amenorrhea (38 vs. 29, *P* < 0.0001) (Figure 4B). Serum AMH (area under ROC 0.77) better discriminated women with oligo/amenorrhea from those with regular menstrual cycles than AFC (area under ROC 0.67), however the combination of the two performed better than either marker alone (area under ROC 0.83; 95% CI 0.77–0.89) (Figure 4C). The odds of oligo/amenorrhea were increased by 6.7-fold (95% CI 2.5–19.9) in those with an AMH per antral follicle of >2 pmol/L when compared with those with an AMH per antral follicle of <1 pmol/L (*P* < 0.0001) (Figure 4D).

## PCOS Diagnosis

In the present cohort, 97 of 182 (53.5%) patients had PCOS by the Rotterdam criteria and 75 of 182 (41.2%) by the new international criteria (2). Menstrual disturbance was present in none of the women without PCOS and 60% of women with PCOS by Rotterdam criteria. Whereas, 8.4% of woman without PCOS and 65% of women with PCOS by the new international criteria had menstrual disturbance. Women with either eumenorrheic or oligo/amenorrheic PCOS had higher serum AMH levels than women without PCOS diagnosed by either Rotterdam criteria or by the new International criteria (Supplementary Table 1). Whilst AMH was higher in women with oligo/amenorrheic PCOS than in women with eumenorrheic PCOS (*P* < 0.009), AFC did not significantly differ (P>0.3) (Supplementary Table 1). Presence of one PCO morphology ovary defined by Rotterdam criteria had 100% sensitivity and 43.7% specificity to diagnose PCOS, as compared with 47.6% sensitivity and 71.0% specificity if PCO was defined by the international criteria.

The strongest predictors for the diagnosis of PCOS were serum AMH and serum testosterone. Despite hyperandrogenemia forming part of the diagnostic criteria of PCOS, its diagnostic performance in identifying PCOS diagnosed by standard means was less than that of AMH. The auROC for testosterone to identify PCOS was 0.70, and auROC for Free Androgen Index (FAI) was 0.71 by Rotterdam criteria. The equivalent figures for auROC for testosterone and FAI if PCOS was diagnosed by the new international criteria was 0.71 and 0.72, respectively. A combination of serum AMH and testosterone identifies the diagnosis of PCOS using the Rotterdam criteria with an area under the ROC of 0.79 (95% CI 0.69–0.86), and of the diagnosis of PCOS by the new international criteria with an area under the ROC of 0.77 (95% CI 0.67–0.85).

### Ovarian Morphology on Ultrasound

Whilst AMH (Supplementary Figure 2A) and FAI (Supplementary Figure 2C) were not significantly different by categories of ovarian morphology, total AFC was higher in women with two ovaries having PCO morphology by Rotterdam criteria (Supplementary Figure 2B). The frequency of oligo/amenorrhea was highest in women with two ovaries demonstrating PCO morphology at 47% (Supplementary Figure 2D). The odds of oligo/amenorrhea were increased by 10.7-fold in women with two PCO morphology ovaries compared to women with normal ovarian morphology (Supplementary Figure 2D).

The distinction between PCO and MCO lies in the amount and distribution of non-follicular stromal tissue (26). Ovarian volume correlates with antral follicle count (Supplementary Figure 3A). Thus, ovarian volume is higher in PCO morphology ovaries than normal morphology ovaries (Supplementary Figure 3B). Therefore, we assessed “ovarian volume per follicle” as an objective surrogate measure of the amount of non-follicular stromal tissue. Ovarian volume per follicle was higher in normal morphology ovaries than MCO ovaries, or PCO ovaries (Supplementary Figure 3C). Women with bilateral polycystic morphology ovaries had lower ovarian volume per follicle than those with normal morphology ovaries (Supplementary Figure 3D). Women with greater AMH levels (Supplementary Figure 3E) had lower ovarian volume per follicle and this was associated with an increased risk of oligo/amenorrhea (Supplementary Figure 3F).

### Relationship Between Body Weight and FAI

**Figure 5:**
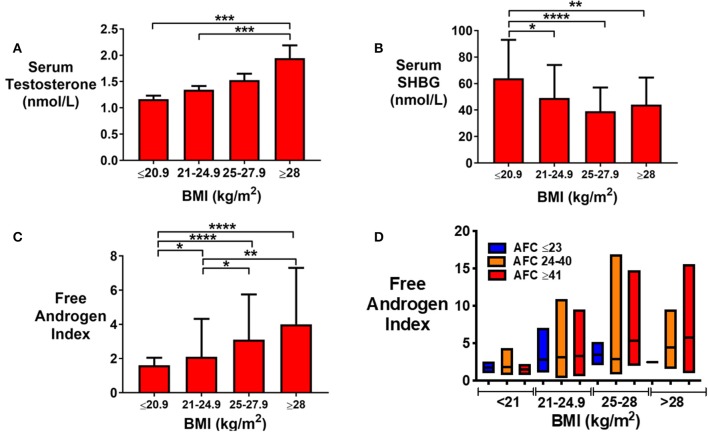
**(A)** Bar graph (median ± IQR) of serum testosterone (nmol/L) by categories of BMI (kg/m^2^). Groups were compared by the Kruskal-Wallis test with *post-hoc* Dunn's multiple comparison test. Serum testosterone was increased by category of BMI. ***P < 0.001. **(B)** Bar graph (median ± IQR) of serum sex hormone binding globulin, SHBG (nmol/L) by categories of BMI (kg/m^2^). Groups were compared by the Kruskal-Wallis test with *post-hoc* Dunn's multiple comparison test. SHBG was reduced in women with higher BMI. *P < 0.05, **P < 0.01, ****P < 0.0001. **(C)** Bar graph (median ± IQR) of Free Androgen Index, FAI by categories of BMI (kg/m^2^). Groups were compared by the Kruskal-Wallis test with *post-hoc* Dunn's multiple comparison test. FAI was increased by BMI category. *P < 0.05, **P < 0.01, ****P < 0.0001. **(D)** Bar graph (median ± IQR) of FAI by categories of BMI (kg/m^2^) stratified by AFC. Median and range is presented. FAI in those with AFC 23 are shown in blue, 24–40 in orange and ≥41 in red.

Serum testosterone was increased (Figure 5A), SHBG level decreased (Figure 5B) and FAI increased (Figure 5C) by category of BMI in non-obese women. There was a trend toward an increase in FAI with BMI even when stratified by AFC (Figure 5D). Body fat mass was the strongest predictor of serum testosterone (*P* < 0.05) followed by AMH and AFC. Using all three to predict log-adjusted testosterone has an *r*^2^ of 15%.

## Discussion

PCOS affects up to a fifth of women of reproductive age and is the commonest cause of anovulatory subfertility (2). Diagnostic criteria for PCOS inevitably transform continuous variables into binary data to facilitate reproducible identification of PCOS. However, many features of PCOS span a spectrum encompassing both health and disease. Furthermore, women with very high AFC are considered no differently to those meeting a threshold value of FNPO to denote PCO morphology. Data from the present study suggests that women with greater increases in ovarian reserve markers, such as AMH or AFC, have an increased risk of menstrual disturbance beyond those with more modest elevations. Additionally, women with more features of PCOS had higher AMH levels than those with fewer features. Moreover, women with higher AMH levels had more LH-predominant gonadotropin secretion consistent with increased GnRH pulsatility. AMH more reliably identified women with PCOS than AFC, but the combination of both ovarian reserve markers better identified PCOS than either marker alone. Overall, our data suggests that AMH has potential as a biomarker for diagnosis of PCOS, outperforming AFC, and greater elevations in AMH were associated with a more certain diagnosis of PCOS.

AMH is secreted by growing antral follicles and thus can be regarded as a surrogate marker of antral follicle count. Locally, AMH plays a paracrine role to inhibit FSH-induced aromatase activity in granulosa cells and aids in the emergence of a dominant follicle (27). However, there is increasing interest in a further putative role for AMH in the pathogenesis of PCOS, acting as an endocrine signal to directly increase GnRH pulsatility (15, 17). Indeed, our data demonstrates that follicular phase gonadotropin levels became more LH-predominant, commensurate with an increase in GnRH pulsatility, in those with greater AMH values. Notably, women with more LH-predominant gonadotropin secretion also had increased rates of menstrual disturbance. Pulse frequency is increased in women with PCOS (healthy controls 16.5, PCOS 22.8 pulse per 24 h), however LH pulse amplitude is reduced in obese (BMI >30 kg/m^2^) women with PCOS, which needs to be considered when interpreting the LH to FSH ratio (28). In our cohort, all women had a BMI < 30 kg/m^2^ and thus gonadotropin secretion was less affected by obesity.

In the present study, the risk of oligo/amenorrhea was gradually increased with serum AMH level rather than the risk being defined by a single threshold. Moreover, there was a trend toward increased cycle length with AMH level in eumenorrheic women in keeping with a recent report (22). Furthermore, whilst AMH levels correlated with AFC, serum AMH better predicted the risk of oligo/amenorrhea than AFC. Accordingly, women with amenorrheic PCOS have been reported to have higher AMH levels than those with oligomenorrheic PCOS, and in turn than eumenorrheic PCOS, and healthy controls (29). Interestingly, in our data the combination of AMH and AFC appeared to improve the prediction of oligo/amenorrhea when compared to each measure alone. Indeed, women with higher AMH per antral follicle had increased odds of oligo/amenorrhea than those with lower values.

Another key feature of PCOS is the presence of hyperandrogenism. Women with elevated BMI have an increased prevalence of PCOS, and thus could also have increased levels of androgens. The prevalence of PCOS is increased by obesity, although not dramatically (BMI <19 kg/m^2^ 8%, BMI 19–30 kg/m^2^ 9.9%, BMI 30–35 kg/m^2^ 5.2%, BMI > 35 kg/m^2^ 11.5–12.4%) (30). However, our data suggested that FAI was more strongly predicted by BMI than by AMH, although women with both increased BMI and high follicle counts were most at risk of biochemical hyperandrogenism (Figure 5D). In keeping with BMI being the strongest predisposing factor, androgen levels have been reported to be higher in women with PCOS with a BMI >25 kg/m^2^ than those with lower BMI's (31, 32). Similarly, FAI, but not total testosterone, were reported to correlate with BMI, both in women with and without PCOS (31, 32). Correspondingly, Penttila et al. have observed that FAI was increased by BMI in healthy controls and in women with PCOS, whether hirsuitism was present or not (32, 33).

Whilst androgen production in PCOS is predominantly of ovarian origin, driven by insulin resistance and exacerbated by BMI, recent data has suggested that other organs, such as adipose tissue can also be a site of androgen production. The androgenic enzyme aldoketoreductase type 3 (AKR1C3) is expressed in subcutaneous adipose tissue and is stimulated by insulin to contribute to hyperandrogenism in obese women (34). Additionally, Deng and colleagues have observed that adrenal-derived androgens may be of particular relevance in lean women with PCOS (35).

Due to the subjective nature of describing PCO morphology based on the appearance of peripherally located follicles around a central stroma (26), PCO morphology is more commonly defined by follicle number per ovary regardless of distribution (2). Multicystic ovarian (MCO) morphology, in which follicle number is increased without the typical distribution, may be associated with hypothalamic amenorrhea, or during adolescence, reflecting hypothalamo-pituitary dysfunction (36–38). In our data, women with PCO morphology in both ovaries had higher AMH, AFC, and rates of menstrual disturbance than in women with at least one ovary with normal ovarian morphology. We strived to objectively quantify the distinction between MCO and PCO morphology by assessing “ovarian volume per antral follicle” (Supplementary Figure 3). Thus, our data is in keeping with the concept that MCO morphology may represent a milder phenotype than those with PCO morphology (39), but definition of ovarian morphology by appearance did not appear to be superior to definition by FNPO.

A number of studies have evaluated the ability of an AMH threshold value to diagnose PCOS. AMH levels were higher in women with PCOS and polycystic ovarian morphology (9.3 mcg/L) than those without PCO morphology alone (6.4 mcg/L), or healthy controls (2.1 mcg/L) (19). AMH was 9.1 mcg/L in women with PCOS and 2.5 mcg/L in controls (40). Similarly, AMH was 5.7 ng/ml in healthy controls, 9.3 ng/ml in women with PCOS and 9.9 ng/ml in women with all three main features of PCOS (41). Using cluster analysis, age was found to be an important modifying variable for AMH to differentiate PCOS from PCO morphology and from healthy women (21). A meta-analysis determined that an AMH of 4.7 ng/ml had a 79.4% sensitivity and 82.8% specificity to identify PCOS (auROC 0.87) (42). In summary, a number of studies suggest that AMH could represent a useful marker for the diagnosis of PCOS, however challenges to the use of AMH to diagnose PCOS are usefully summarized in Teede et al. (43). Outstanding areas of research include the need for data using standardized optimal assays, to associate thresholds with clinical features of PCOS, and to stratify reference ranges by age (43). Pigny et al. (11) compared the performance of five commercial AMH assays in the diagnosis of PCOS and found that whilst the assays offered similar performance, newer automated assays reported 23–30% lower values than manual assays (11). Our data is in keeping with the view that women with PCOS have a spectrum of phenotypic presentation that can be altered by baseline variables, such as age or BMI e.g., women with PCOS are less likely to have oligo/amenorrhea in older years (44). Thus, the construct of risk-models using continuous variables to quantify the likelihood of PCOS from an unselected population would be of value, although this remains challenged by the lack of an objective gold standard test to diagnose PCOS.

Strengths of our data include that women were routinely screened for features of PCOS by a specialist unit with experience in the accurate assessment of AFC and ovarian morphology with a limited number of sonographers conducting assessments, thus reducing inter-observer bias. Limitations of our data include that women recruited to the study were not an unselected group of women, but rather young women seeking fertility treatment. Consequently, women over 35 years, with low AMH < 10 pmol/L, or obesity, were not included in the present study. Furthermore, it was not possible to address whether higher values of AMH over the reporting limit of the assay would confer even greater risk of menstrual disturbance. Thus, further prospective research in an unselected population with newer automated assays is indicated to determine whether the value of an AMH level in predicting menstrual disturbance is underestimated in the current study (11).

In conclusion, our data suggests that AMH is a strong predictor for menstrual disturbance due to PCOS and that the risk of menstrual disturbance was increased with the degree of elevation of AMH. AMH performed better than AFC in predicting menstrual disturbance, although the combination of both parameters outperformed AMH alone. Furthermore, women with a greater number of features of PCOS had higher AMH levels than those with fewer features, suggesting that AMH could reflect the certainty of diagnosis. Our data suggests that AMH is a promising marker for the identification of menstrual disturbance due to PCOS.

## Data Availability Statement

All datasets generated for this study are included in the manuscript/Supplementary Files.

## Ethics Statement

The studies involving human participants were reviewed and approved by Hammersmith and Queen Charlotte's Research Ethics Committee (reference: 10/H0707/2). The patients/participants provided their written informed consent to participate in this study.

## Author Contributions

AA, GC, AC, GT, RS, and WD designed the study. Data were collected by AA, PE, MP, SC, TH, RR, SV, RI, KP, and AC. Analysis was carried out by AA, PE, MP, TH, AH, and TK. LO provided support in the writing of this manuscript. WD took the final responsibility for this article. All authors provided contributions to study conception and design, acquisition of data or analysis and interpretation of data, drafting the article or revising it critically for important intellectual content, and final approval of the version to be published.

### Conflict of Interest

The authors declare that the research was conducted in the absence of any commercial or financial relationships that could be construed as a potential conflict of interest.

## Supplementary Material

Supplementary Figure 1 **(A)** Scattergram (median ± IQR) of serum AMH (pmol/L) in women by the number of features of PCOS (new PCOS criteria). Groups were compared by the Kruskal Wallis test with *post-hoc* Dunn's multiple comparison test. Serum AMH was increased by the number of features of PCOS. *****P* < 0.0001. **(B)** Scattergram (median ± IQR) of AFC in women by the number of features of PCOS (new PCOS criteria). Groups were compared by Kruskal Wallis test with *post-hoc* Dunn's multiple comparison test. AFC was increased by the number of features of PCOS. **P* < 0.05, *****P* < 0.0001. **(C)** Scattergram (median ± IQR) of FAI in women by the number of features of PCOS (new PCOS criteria). Groups were compared by the Kruskal Wallis test with *post-hoc* Dunn's multiple comparison test. FAI was increased by the number of features of PCOS. **P* < 0.05, ***P* < 0.01. **(D)** Scattergram (median ± IQR) of BMI (kg/m^2^) in women by number of features of PCOS based on new criteria. Groups were compared by the Kruskal Wallis test with *post-hoc* Dunn's multiple comparisons test. Women with average menstrual cycle length of ≥35 days have higher AMH levels compared to those with menstrual cycle length of ≤ 27 days (*P* < 0.0001) or those with menstrual cycle length of 28–30 days. **P* < 0.05, ***P* < 0.01, ****P* < 0.001.

Supplementary Figure 2 **(A)** Scattergram (median ± IQR) of serum AMH (pmol/L) by sonographic morphology of each ovary as either Normal (Norm), Multicystic (MCO), or Polycystic (PCO). The sample size in each group was as follows: Norm Norm (*n* = 26); Norm MCO (*n* = 9); MCO MCO (*n* = 18); MCO PCO (*n* = 5); Norm PCO (*n* = 10); PCO PCO (*n* = 119). There were no significant differences between groups. **(B)** Scattergram (median ± IQR) of AFC by sonographic morphology of ovaries. Comparison was made by the Kruskal Wallis test with Tukey's multiple comparison test. Women with bilateral PCO morphology had higher AFC than those with bilateral Normal morphology (*P* = 0.016) or bilateral MCO morphology (*P* < 0.0001). ****P* < 0.001, *****P* < 0.0001. **(C)** Scattergram (median ± IQR) of Free Androgen Index (FAI) by sonographic morphology of ovaries. **(D)** Frequency of oligomenorrhea was increased in women with polycystic ovarian morphology (PCO) by univariate logistic regression (*P* < 0.001). The odds of oligomenorrhea was increased by 1.5-fold (95% CI 0.19–11.8) in women with bilateral multicystic ovarian morphology (MCO-MCO) and 10.7-fold (95% CI 2.4–47.1) in women with bilateral polycystic ovarian (PCO-PCO) morphology in comparison to those with normal ovarian morphology.

Supplementary Figure 3 **(A)** Total Antral Follicle Count (AFC) was associated with Total Ovarian Volume (ml) (sum of left and right ovarian volumes; *r*^2^ = 0.25, *P* < 0.0001). **(B)** Scattergram (median ± IQR) of the volume of each ovary (mls) by its morphology: either normal, multicystic (MCO), or polycystic (PCO). Groups were compared by the Kruskal-Wallis test with *post-hoc* Dunn's multiple comparison test. Ovarian volume was greater in PCO morphology ovaries than normal morphology ovaries. *****P* < 0.0001. **(C)** Scattergram (median ± IQR) of ovarian volume per antral follicle (mls) (ovarian volume divided by antral follicle count in each ovary) by categories of morphology of that ovary. Groups were compared by the Kruskal-Wallis test with *post-hoc* Dunn's multiple comparison test. Ovarian volume per antral follicle was lower in PCO ovaries than normal morphology ovaries. *****P* < 0.0001. **(D)** Scattergram (median ± IQR) of ovarian volume per antral follicle (mls) (i.e., total ovarian volume divided by total antral follicle count from both ovaries) by categories of ovarian morphology. Groups were compared by Kruskal-Wallis with *post-hoc* Dunn's multiple comparison test. Ovarian volume per antral follicle was reduced in women with bilateral PCO morphology in comparison to those with bilateral normal ovarian morphology. **P* < 0.05, *****P* < 0.0001. **(E)** Bar graph (mean ± SD) of ovarian volume per antral follicle (mls) by categories of serum AMH (pmol/L). Groups were compared by the Kruskal-Wallis with *post-hoc* Dunn's multiple comparison test. Ovarian volume per antral follicle was lower in women with higher serum AMH. **P* < 0.05, ***P* < 0.01. **(F)** Frequency of oligomenorrhea decreased with increased category of ovarian volume per antral follicle (mls). Groups were compared by univariate logistic regression. The odds of oligo/amenorrhea was reduced in women with increased ovarian volume per antral follicle when compared with those with ovarian volume per antral follicle 0–0.25; ovarian volume per antral follicle 0.26–0.50; OR 0.15 (95% CI 0.04–0.60), 0.51–0.75; OR 0.13 (95% CI 0.03–0.56), 0.76–1.00; OR 0.10 (95% CI 0.02–0.54), >1.00; OR 0.03 (95% CI 0.01–0.34).

Supplementary Table 1 Median and interquartile range (IQR) of serum anti-Mullerian hormone (AMH) and of total antral follicle count (AFC) are presented. Five women were not included in this analysis due to missing data. Serum AMH and total antral follicle counts (AFC) in eumenorrheic and oligo/amenorrheic women were compared by the Mann-Whitney *U* test.
