# Supplementary figures and images for: Anti-Müllerian hormone (AMH) in the Diagnosis of Menstrual Disturbance Due to Polycystic Ovarian Syndrome
Source: Front Endocrinol (Lausanne). 2019 Sep 26;10:656. doi: 10.3389/fendo.2019.00656 (PMC6775233; doi:10.3389/fendo.2019.00656)

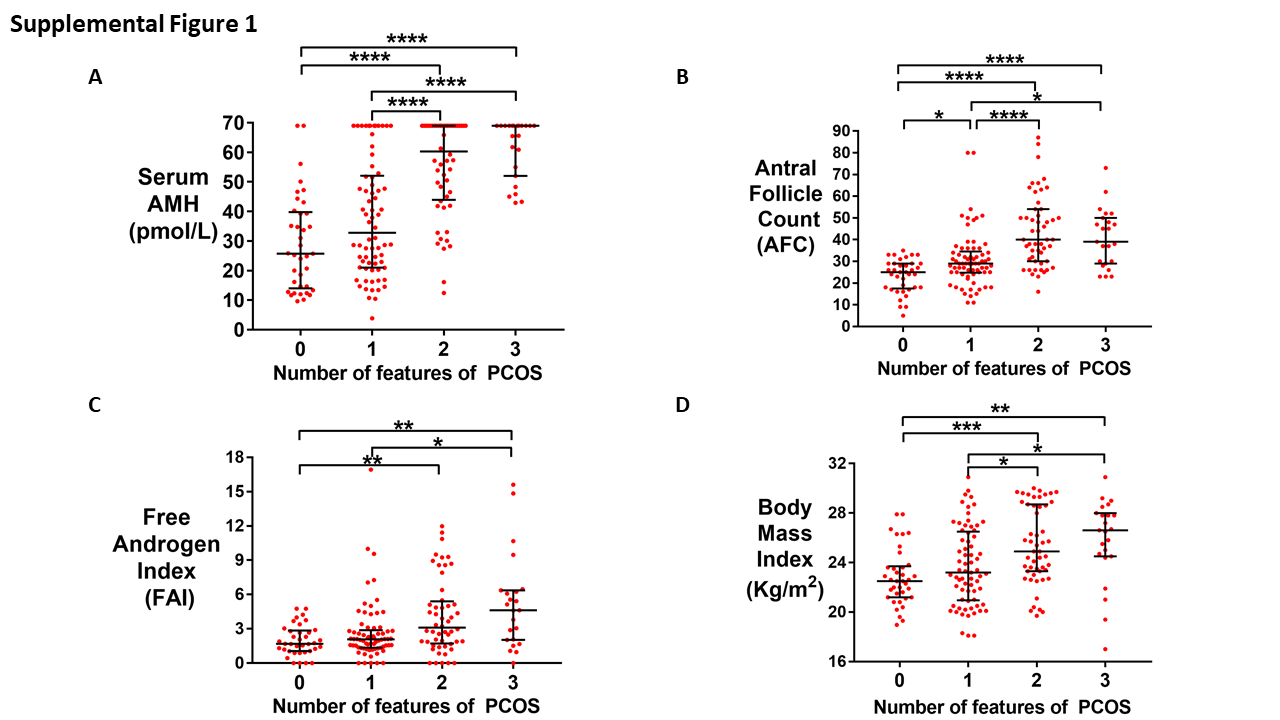

Supplement: Supplementary Figure 1 — (A) Scattergram (median ± IQR) of serum AMH (pmol/L) in women by the number of features of PCOS (new PCOS criteria). Groups were compared by the Kruskal Wallis test with post-hoc Dunn's multiple comparison test. Serum AMH was increased by the number of features of PCOS. ****P < 0.0001. (B) Scattergram (median ± IQR) of AFC in women by the number of features of PCOS (new PCOS criteria). Groups were compared by Kruskal Wallis test with post-hoc Dunn's multiple comparison test. AFC was increased by the number of features of PCOS. *P < 0.05, ****P < 0.0001. (C) Scattergram (median ± IQR) of FAI in women by the number of features of PCOS (new PCOS criteria). Groups were compared by the Kruskal Wallis test with post-hoc Dunn's multiple comparison test. FAI was increased by the number of features of PCOS. *P < 0.05, **P < 0.01. (D) Scattergram (median ± IQR) of BMI (kg/m2) in women by number of features of PCOS based on new criteria. Groups were compared by the Kruskal Wallis test with post-hoc Dunn's multiple comparisons test. Women with average menstrual cycle length of ≥35 days have higher AMH levels compared to those with menstrual cycle length of ≤ 27 days (P < 0.0001) or those with menstrual cycle length of 28–30 days. *P < 0.05, **P < 0.01, ***P < 0.001. [file Image_1.TIF]

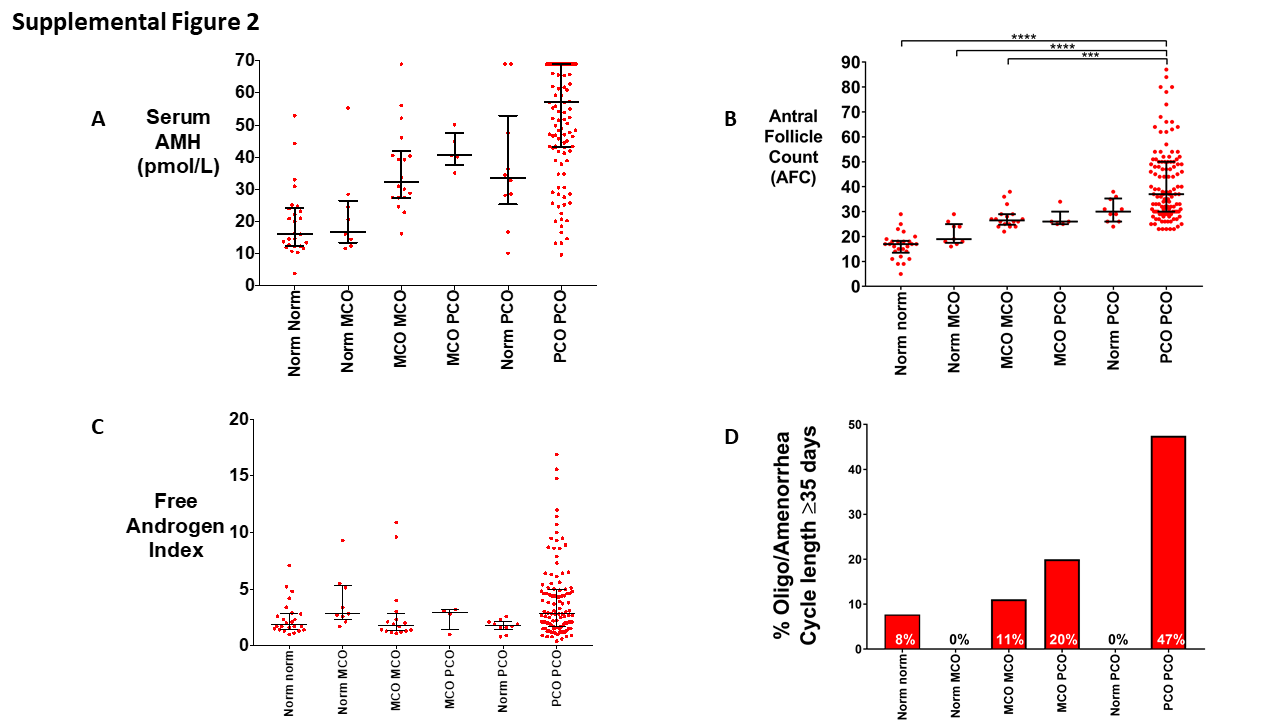

Supplement: Supplementary Figure 2 — (A) Scattergram (median ± IQR) of serum AMH (pmol/L) by sonographic morphology of each ovary as either Normal (Norm), Multicystic (MCO), or Polycystic (PCO). The sample size in each group was as follows: Norm Norm (n = 26); Norm MCO (n = 9); MCO MCO (n = 18); MCO PCO (n = 5); Norm PCO (n = 10); PCO PCO (n = 119). There were no significant differences between groups. (B) Scattergram (median ± IQR) of AFC by sonographic morphology of ovaries. Comparison was made by the Kruskal Wallis test with Tukey's multiple comparison test. Women with bilateral PCO morphology had higher AFC than those with bilateral Normal morphology (P = 0.016) or bilateral MCO morphology (P < 0.0001). ***P < 0.001, ****P < 0.0001. (C) Scattergram (median ± IQR) of Free Androgen Index (FAI) by sonographic morphology of ovaries. (D) Frequency of oligomenorrhea was increased in women with polycystic ovarian morphology (PCO) by univariate logistic regression (P < 0.001). The odds of oligomenorrhea was increased by 1.5-fold (95% CI 0.19–11.8) in women with bilateral multicystic ovarian morphology (MCO-MCO) and 10.7-fold (95% CI 2.4–47.1) in women with bilateral polycystic ovarian (PCO-PCO) morphology in comparison to those with normal ovarian morphology. [file Image_2.TIF]

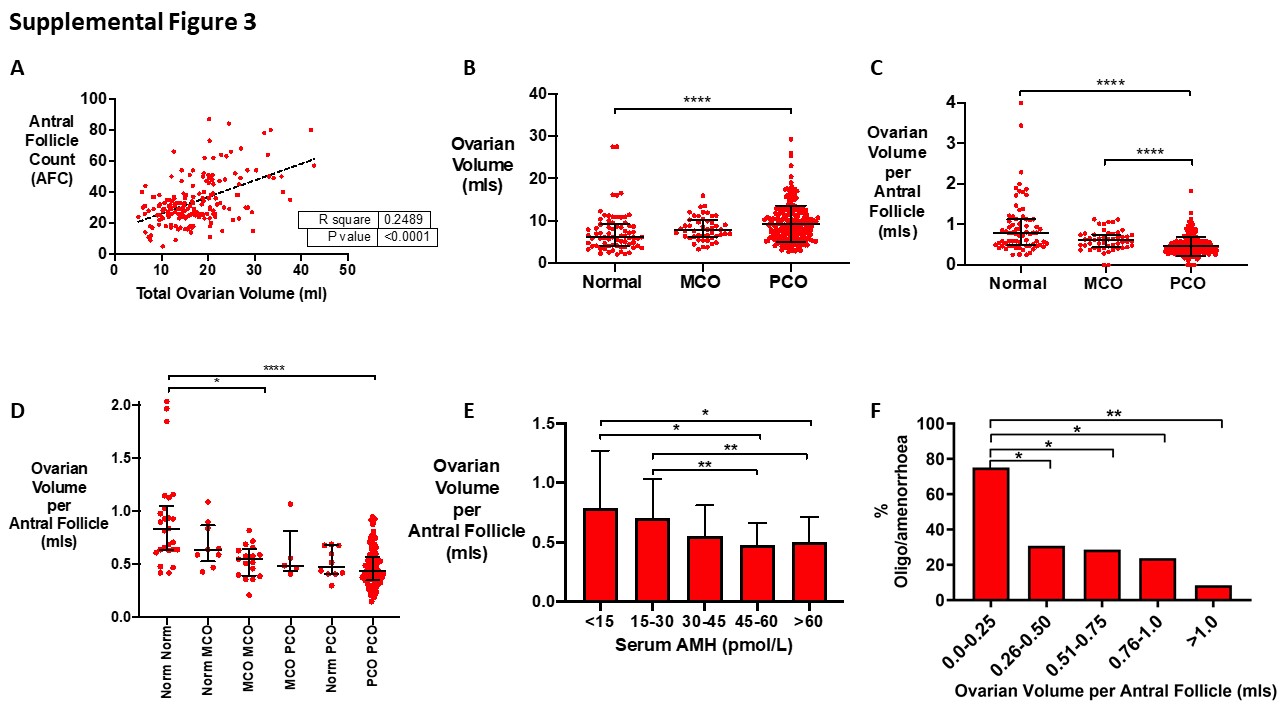

Supplement: Supplementary Figure 3 — (A) Total Antral Follicle Count (AFC) was associated with Total Ovarian Volume (ml) (sum of left and right ovarian volumes; r2 = 0.25, P < 0.0001). (B) Scattergram (median ± IQR) of the volume of each ovary (mls) by its morphology: either normal, multicystic (MCO), or polycystic (PCO). Groups were compared by the Kruskal-Wallis test with post-hoc Dunn's multiple comparison test. Ovarian volume was greater in PCO morphology ovaries than normal morphology ovaries. ****P < 0.0001. (C) Scattergram (median ± IQR) of ovarian volume per antral follicle (mls) (ovarian volume divided by antral follicle count in each ovary) by categories of morphology of that ovary. Groups were compared by the Kruskal-Wallis test with post-hoc Dunn's multiple comparison test. Ovarian volume per antral follicle was lower in PCO ovaries than normal morphology ovaries. ****P < 0.0001. (D) Scattergram (median ± IQR) of ovarian volume per antral follicle (mls) (i.e., total ovarian volume divided by total antral follicle count from both ovaries) by categories of ovarian morphology. Groups were compared by Kruskal-Wallis with post-hoc Dunn's multiple comparison test. Ovarian volume per antral follicle was reduced in women with bilateral PCO morphology in comparison to those with bilateral normal ovarian morphology. *P < 0.05, ****P < 0.0001. (E) Bar graph (mean ± SD) of ovarian volume per antral follicle (mls) by categories of serum AMH (pmol/L). Groups were compared by the Kruskal-Wallis with post-hoc Dunn's multiple comparison test. Ovarian volume per antral follicle was lower in women with higher serum AMH. *P < 0.05, **P < 0.01. (F) Frequency of oligomenorrhea decreased with increased category of ovarian volume per antral follicle (mls). Groups were compared by univariate logistic regression. The odds of oligo/amenorrhea was reduced in women with increased ovarian volume per antral follicle when compared with those with ovarian volume per antral follicle 0–0.25; ovarian volume per antral follicl [file Image_3.jpg]
