# Supplementary material for: Anti-Müllerian hormone (AMH) in the Diagnosis of Menstrual Disturbance Due to Polycystic Ovarian Syndrome
Source: Front Endocrinol (Lausanne). 2019 Sep 26;10:656. doi: 10.3389/fendo.2019.00656 (PMC6775233; doi:10.3389/fendo.2019.00656)
Supplement: Supplementary Table 1 — Median and interquartile range (IQR) of serum anti-Mullerian hormone (AMH) and of total antral follicle count (AFC) are presented. Five women were not included in this analysis due to missing data. Serum AMH and total antral follicle counts (AFC) in eumenorrheic and oligo/amenorrheic women were compared by the Mann-Whitney U test. [file Table_1.docx]

**Supplemental Table 1:**

|  | Women without PCOS | Eumenorrheic PCOS | Oligo/Amenorrheic PCOS | P Value  Eumenorrheic vs Oligo/Amenorrheic PCOS |
| --- | --- | --- | --- | --- |
| Rotterdam Criteria | N=85 | N=39 | N=58 |  |
| Serum AMH  pmol/L  Median (IQR) | 25.8  (16.5, 68.9) | 50.5  (32.8, 68.9) | 67.4  (45.6, 68.9) | 0.0072 |
| Antral Follicle Count  (AFC)  Median (IQR) | 27.0  (18.0, 33.0) | 33.0  (27.0, 44.0) | 37.0  (28.0, 50.5) | 0.30 |
|  |  |  |  |  |
| 2018 International Criteria | N=107 | N=25 | N=50 |  |
| Serum AMH  pmol/L  Median (IQR) | 28.8  (20.0, 47.2) | 52.4  (31.7, 68.9) | 68.9  (53.4, 68.9) | 0.0089 |
| Antral Follicle Count  (AFC)  Median (IQR) | 27.0  (22.0, 32.0) | 39.0  (31.5, 49.3) | 42.5  (29.3, 52.0) | 0.78 |

Median and interquartile range (IQR) of serum anti-Mullerian hormone (AMH) and of total antral follicle count (AFC) are presented. Five women were not included in this analysis due to missing data. Serum AMH and total antral follicle counts (AFC) in eumenorrheic and oligo/amenorrheic women were compared by the Mann Whitney U test.
